# The Crosstalk between the Gut Microbiota Composition and the Clinical Course of Allergic Rhinitis: The Use of Probiotics, Prebiotics and Bacterial Lysates in the Treatment of Allergic Rhinitis

**DOI:** 10.3390/nu14204328

**Published:** 2022-10-16

**Authors:** Agnieszka Kaczynska, Martyna Klosinska, Paulina Chmiel, Kamil Janeczek, Andrzej Emeryk

**Affiliations:** Department of Pulmonary Diseases and Children Rheumatology, Medical University of Lublin, 6 Profesora Antoniego Gębali Street, 20-093 Lublin, Poland

**Keywords:** allergic rhinitis, allergy, gut microbiota, probiotics, prebiotics, bacterial lysates, faecal microbiota transplantation

## Abstract

Although massive progress in discovering allergic rhinitis (AR) aetiology has been made in recent years, its prevalence is still rising and it significantly impacts patients’ lives. That is why further and non-conventional research elucidating the role of new factors in AR pathogenesis is needed, facilitating discoveries of new treatment approaches. One of these factors is the gut microbiota, with its specific roles in health and disease. This review presents the process of gut microbiota development, especially in early life, focusing on its impact on the immune system. It emphasizes the link between the gut microbiota composition and immune changes involved in AR development. Specifically, it elucidates the significant link between bacteria colonizing the gut and the Th1/Th2 imbalance. Probiotics, prebiotics and bacterial lysates, which are medications that restore the composition of intestinal bacteria and indirectly affect the clinical course of AR, are also discussed.

## 1. Introduction

**Figure 1:**
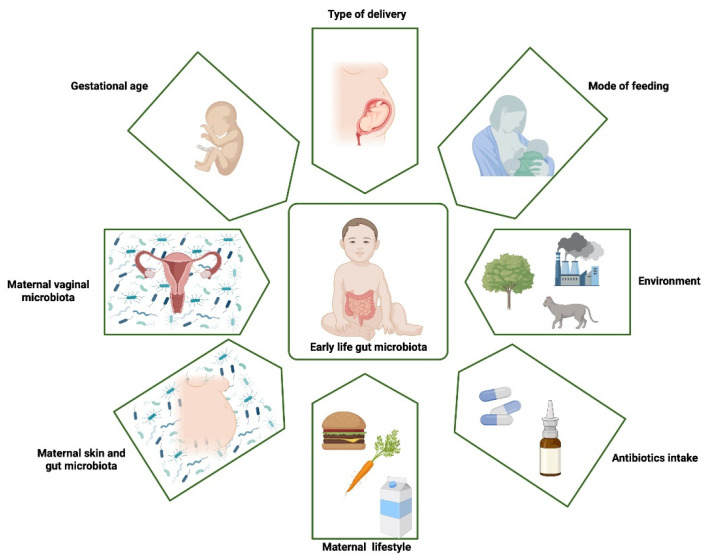
The main factors that influence early-life gut microbiota. The first steps in gut colonization probably start already in utero and are influenced by maternal gut microbiota. More likely, a new-born is specifically colonized during birth, and the composition of bacteria localized in the intestine depends on the type of delivery. In the first two years of life, the gut microbiota composition is influenced by multiple factors, mainly mode of feeding, environment and antibiotics intake. Created with biorender.com, accessed on 24 September 2022.

The human organism is not a sterile, closed system. Multiple microorganisms implant on its surfaces and interiors and develop into the local microbiota. The main places with bacterial presence are the gut, skin, and urogenital and respiratory tracts [1]. Figure 1 illustrates the crucial factors that influence gut microbiota development. In recent years, plenty of information has been compiled about microbiota’s significant impact on human health. Its ability to modulate immune system response remains one of the most popular issues of 21st century science.

A disturbance of the immune system is the cause of multiple diseases, including allergic ones [2]. Speculations on their development centre around innate and adaptive immune response abnormalities. Firstly, the hygiene hypothesis enlightens the importance of the proper innate response maturation. Secondly, the imbalance of Th1/Th2 responses seems to play a significant role in the pathogenesis of allergic diseases [3].

Allergic rhinitis (AR) is a widely prevalent condition of the upper respiratory tract. It affects mainly children with up to 40% of the population worldwide [4]. It is characterized by rhinorrhoea, sneezing, and a blocked and itchy nose [5]. Such symptoms affect patients’ quality of life and lead to multiple nuisances such as sleep disturbances, learning disabilities and changes in behaviour. Furthermore, AR promotes sinusitis, otitis and asthma exacerbations [6,7]. The treatment of AR is based mainly on nasal corticosteroids as well as nasal and oral H1-antihistamines; in addition, anticholinergic, antileukotriene and alpha-mimetic agents and chromones are used. However, the effectiveness of these medications is limited, and they are burdened with multiple side effects [8].

As mentioned before, the immune system is constantly stimulated by the plethora of ligands presented by bacteria colonizing the intestine, such as lipopolysaccharides, unmethylated CpG motifs, fatty acids and flagellin [9]. They are recognized by Toll-like receptors (TLRs) and stimulate the immune system. It shapes the differentiation of naïve T cells into Tregs, Th1, Th2 and Th17; modifies the levels of eosinophils, basophils and mast cells; and influences the production of IgE [10]. Considering that these processes are closely related to the composition of the gut microbiota, its significant role in the pathogenesis of allergic disorders remains valid [11].

This review aims to summarize the interplay between the gut microbiota and AR’s development and clinical course. Furthermore, immunomodulatory preparations that alter the composition of microbes present in the intestine and their effects in AR treatment are also discussed.

## 2. Gut Microbiota Development and the Risk of Allergic Rhinitis

### 2.1. Prenatal Period

The exact time of gut colonization has yet to become known. The ‘sterile womb paradigm’ has been the accepted dogma for years. It was believed that microbial implementation starts during or right after birth and depends on the delivery type [12]. However, at the end of the 20th century, the research on gut microbiota gathered pace, and some studies have proposed that not all healthy new-borns are born sterile. The microorganisms were found in the amniotic fluid [13,14], umbilical cord [15], placenta [14], and meconium [16,17]. In animal studies, bacteria in the foetal gut resembled those from the maternal intestine [16]. It can be suspected that multiple hormonal changes during the pregnancy provoke a transfer of bacteria from the intestinal epithelium to the placenta. 

Nevertheless, maternal gut microbiota more likely produces compounds that affect the developing immune system of the foetus. Thorburn et al. showed that feeding pregnant mice a high-fibre diet yielded a distinctive gut microbiota, which increased the levels of the short-chain fatty acid (SCFA) acetate. It reduced the symptoms of allergic airways disease in cubs by enhancing Tregs number and function [18]. Furthermore, Venter et al. developed a maternal diet index during pregnancy that was associated with offspring allergy outcomes. Data suggest that high vegetables and yoghurt intake, which have been reported to increase the gut microbiome diversity, reduce the risk of AR, atopic dermatitis (AD) and asthma [19]. Notably, this microbiome diversity promotes higher levels of faecal butyrate, an SCFA that stimulates the development of Tregs and promotes macrophage differentiation [20,21]. Nevertheless, foods high in advanced glycosylated end-products reduce the diversity of bacteria present in the maternal intestine, which minimizes the production of metabolites favourable in foetal immune development [22]. Furthermore, not only diet during pregnancy has an impact on the gut microbiota composition. Numerous medications alter the microbial diversity and modulate the immature foetal immune system. In a mouse model, prenatal antibiotic exposure significantly altered new-borns’ gut microbiota composition. The treatment decreased the presence of *Bacteroides* spp. and *Firmicutes* spp. and significantly increased the relative abundance of *Proteobacteria* spp. in the intestine compared with the control group [23]. Remarkably, *Bacteroides* spp. secrete a polysaccharide that promotes the differentiation of Tregs and influences the Th1/Th2 balance [24].

### 2.2. Birth Mode

As mentioned in the previous paragraph, the mode of birth for years was considered to be the main factor influencing the gut microbiota composition. Indeed, during vaginal birth, an infant is exposed to microbes from the maternal intestine, urogenital tract and skin, while a Caesarean section provokes colonization mainly by bacteria associated with the skin, mouth and hospital environment [25]. Notably, not all microbes can colonize a new-born’s gut. Microbiota compositions differ among children born in different ways until the end of the first year of life. The intestine of infants born vaginally presents a greater concentration of *Bacteroides* spp., *Bifidobacteria* spp., and *Lactobacillus* spp. On the other hand, the microbiome of new-borns delivered by Caesarean section is composed mainly of *Staphylococcus* spp., *Streptococcus* spp., and *Clostridium* spp. [26]. Of note, bacteria species have their own abilities to modulate the immune system. For instance, Pang et al. showed that *Bacteroides* spp. diminishes the secretion of Th2 cytokines (IL-4, IL-5 and IL-13) and activates Tregs, which ameliorates allergic airway inflammation [27]. Furthermore, *Lactobacillus* spp. promotes the maintenance of Th1/Th2 balance, decreases levels of proinflammatory cytokines (IL-6 and TNF-alfa) and inhibits IgE secretion [28]. On the other hand, *Staphylococcus aureus* enterotoxins act as superantigens and promote Th2 type immune response, IgE secretion and eosinophilic inflammation [29]. These interplays seem to validate the belief that the risk of allergy is enhanced in children born by Caesarean section. Nevertheless, appropriate maturation of the gut microbiota in the first year of life could mitigate these abnormalities [30].

### 2.3. Early Childhood

Many factors impact the acquisition and evolution of gut microbiota in early childhood. Among them, breastfeeding status seems to be the most influential. Breast milk is the most recommended first source of nutrition. It supports growth and development and provides passive immunity to protect against pathogens [31]. Moreover, a specific composition of human milk significantly impacts bacteria species colonizing the foetal gut [32]. Breastfed infants have a specific high amount of *Bifidobacterium* spp., a genus known for its numerous health benefits. Furthermore, exclusively breastfed children have increased taxa levels used as probiotics such as *Lactobacillus johnsonii, paracasei/casei* and *Bifidobacterium longum*. However, formula-fed infants’ gut is markedly similar to that of older children and colonized by *Clostridium difficile, Granulicatella adiacens, Citrobacter* spp. and *Enterobacter* spp. in tremendous amount [33].

*Bifidobacterium* and *Lactobacillus* spp. in human milk activate IgA producing plasma cells in the neonatal gut [34]. Furthermore, they are noted to control local inflammation by mucosal host–microbiota crosstalk and are associated with a lower risk of allergy later in life [35].

Interestingly, a short exposure to antibiotics, especially in the first two years of life, can shift the gut microbiota to long-term dysbiosis. Loss of diversity or specific important taxa, shifts in metabolic capacity and reduced resistance against pathogens may indicate post-antibiotic dysbiosis [36]. Notably, the overgrowth of *Enterobacteriaceae* and reduced diversity of *Firmicutes* and *Bacteroidetes* are typical of the described phenomenon [37]. These disturbances increase the host’s vulnerability to the invasion of *Clostridium difficile* [38] and vancomycin-resistant *Enterococcus* [39]. The retrospective cohort study by Mitre et al. showed a significant link between antibiotics’ treatment in infancy and development of allergic disease. For instance, allergic rhinitis was increased by 75% in children exposed to antibiotics [40].

Studies suggest that the perturbations in the intestinal microbes’ composition may increase the risk of allergy [1,11,23,33,41]. However, the microbiota status in early life seems to be the most important in the immune system development. For instance, neonatal mice treated with streptomycin and vancomycin had a reduced diversity of gut microbiota and an increased risk of asthma development. However, similar effects were not observed in adult mice [41]. It is presumed that similar phenomena can be observed in humans. 

## 3. Gut Microbiota Composition in Health and in Allergic Rhinitis

The intestinal microbiota contains more than 1500 bacteria species [42]. Some bacteria species are typical for all healthy individuals. *Firmicutes, Bacteroidetes, Proteobacteria* and *Actinobacteria* make up to 90% of the total microbial population [43]. Remarkably, altered gut diversity is more common in patients suffering from allergic diseases. Liu et al. enrolled 93 AR patients and 72 healthy controls (HCs) and analysed their gut microbiota composition. Research showed that AR patients had a significantly lower microbiota diversity with an increased abundance of *Bacteroidetes* and lower levels of *Actinobacteria* and *Proteobacteria* than HCs [44]. Similar findings were shown by Watts et al. and Zhu et al., who also pointed out the reduced abundance of *Clostridiales* in AR patients [45,46]. Interestingly, not all allergic diseases are characterized by the same bacteria species colonizing the gut. The microbiota differs substantially between patients with AD, chronic urticaria and AR, which indicates that intestinal bacteria colonies differ significantly between patients with allergic skin disease and allergic nasal disease. Furthermore, it validates the existence of the gut–skin and gut–nose axes [47]. 

## 4. Shaping the Immune System by Gut Microbiota

**Figure 2:**
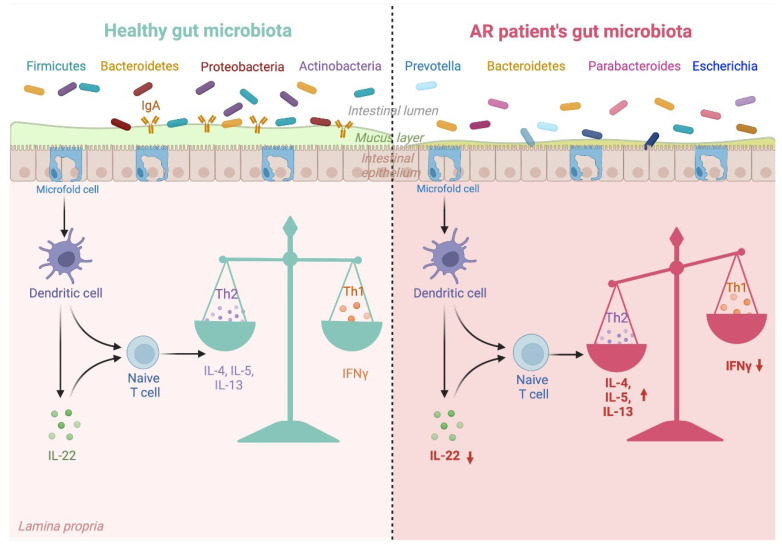
Gut microbiota dysbiosis in AR patients and its impact on Th1/Th2 balance. Gut microbiota composition differs between healthy individuals and AR patients. Gut microbiota typical for AR promotes unfavourable changes in cytokines, which promote the Th1/Th2 imbalance involved in AR development. Created with biorender.com, accessed on 24 September 2022.

As mentioned before, the gut microbiota acts as a new organ that influences the development of the immune system. The term “gut–organ axis” points to the significant crosstalk between the bacteria species colonizing the intestine and processes taking place in the nose, lungs, brain and skin. The microbiota of a healthy infant born vaginally and fed with breast milk correctly shapes its immune system. However, every disturbance of gut composition can negatively influence immature immunity and disrupt innate and adaptive response. Figure 2 explains the link between altered gut microbiota composition and its impact on the immune system in AR patients. 

The intestinal epithelial cells (IECs; microfold cells) bridge the bacteria and the host’s immune system. They translate the commensal bacteria-derived signals (bacterial metabolites, bacterial components, and bacteria themselves) and send them to mucosal immune cells. Such crosstalk, where IECs play a crucial role in intestinal immunity, was seen in germ-free mice colonized with segmented filamentous bacteria. The microbes colonized the intestine and, via IECs, induced the production of serum amyloid A, which improved the Th17 differentiation and IL-22 production [48].

The immune cells mainly engaged in the crosstalk with bacteria colonizing the intestine are primarily seen in the lamina propria. Among them, the most common are dendritic cells (DCs), Tregs, NK cells and CD4+ T cells.

DCs have a crucial role in interacting with innate and adaptive immune responses. They migrate to secondary lymphoid tissues and stimulate CD4+ T cells to differentiate into subtypes based on the activation signal. The commensal bacteria-derived metabolites influence the DCs’ functions. For instance, SCFAs suppress IL-12 and increase IL-10 and IL-23 production [49,50]. Furthermore, they decrease the levels of CCL3, CCL4, CCL5, CXCL9, CXCL10 and CXCL11, indirectly regulating T cell functions [51]. Moreover, they induce B-cell IgA class switching and IgA production and regulate other adaptive response cell functions via DCs’ modulation [52]. 

Macrophages associated with the gut epithelium have a broad spectrum of functions. They can ingest pathogens, produce multiple cytokines that affect other immune cells and support the maintenance of Tregs. Liu et al. showed that SCFAs produced by microbes promote anti-inflammatory IL-10 secretion by macrophages [53]. Furthermore, they shift macrophages’ metabolism, reduce mTOR kinase activity and increase anti-microbial peptide production [54].

In this review, the interplay between the gut microbiota and T cells is limited to the microbes’ impact on the Th1/Th2 balance and its significant role in AR development. Notably, in early life, systemic immune responses are biased toward Th2 [55]. Therefore, the proper composition of bacteria in the intestine is an issue that provides mentioned balance maintenance. Qian et al. investigated the effect of various early life exposures on gut microbial colonization in mice. The research showed that diversity of the intestinal flora in early life influences the levels of IL-4 and IFN-γ, which may prevent airway inflammation in asthma via regulating the Th1/Th2 balance [56]. Furthermore, Jakobsson et al. pointed out that lower microbial diversity during the first two years of life delays the colonization of *Bacteroidetes* and results in reduced Th1-type response [12].

Type 2 innate lymphoid cells (ILC2) are innate immune cells that are deprived of surface markers, which makes them difficult to identify. They mirror the Th2 type cells and have a considerable role in allergy development [57]. Notably, the gut microbiota impacts the migration of ILC2s from the gut to the lung through the gut–lung axis. For instance, *Proteobacteria* significantly facilitate said migration and promote the production of IL-33 [58]. Furthermore, Chua et al. associated the development of respiratory allergies with the increased abundance of *Ruminococcus gnavus*. They showed that intestinal dysbiosis stimulated ILC2s and DCs to produce type 2 cytokines and promoted lung infiltration by eosinophils and mastocytes [59]. On the other hand, SCFAs derived from fermentation of dietary fibres by the gut microbiota inhibit the function of ILC2s and prevent lung inflammation [60]. Thus, studies suggest that the activity of ILC2s is modulated by the gut microbiota, but its underlying mechanisms are still insufficiently elucidated.

## 5. Preparations Affecting Gut Microbiota Composition and Their Effects in Allergic Rhinitis Treatment

With rising allergy prevalence, knowledge of microbiota situated in the gastrointestinal tract and its beneficial effects becomes increasingly important. On the one hand, probiotics, prebiotics and symbiotics affect the gut microbiota composition by counteracting the activity of undesirable bacteria and by modulating the host’s metabolism. On the other hand, bacterial lysates (BLs) indirectly influence the gut environment by stimulating DCs in the gut mucosa and by modulating the immune response. Finally, foecal microbiota transplantation (FMT), as a novel therapy, is likely to ensure stable gut microbiota maintenance.

### 5.1. Probiotics

The World Health Organization defines probiotics as “live strains of strictly selected microorganisms which, when administered in adequate amounts, confer a health benefit on the host” [61]. Noticeably, not all beneficial bacteria species can meet the requirements to become probiotics. The safety of the bacteria strain is defined by its origin, the absence of pathogenicity, and the antibiotic resistance profile. Moreover, probiotics should be easy to produce and should easily maintain their properties through the distribution process [62].

Probiotic products contain of one or more bacteria strains. The most used are *Lactobacillus, Bifidobacterium, Lactococcus, Streptococcus* and *Enterococcus* [63]. Their main advantage is the ability to ensure a proper balance between microorganisms that impact human’s health. They promote antagonism through the production of antimicrobial products, compete with pathogens, inhibit bacteria toxin production and modulate the host’s immune system [64].

Several human studies have evaluated the efficacy of probiotics in the prevention and treatment of allergic diseases, including AR. The first clinical trial that examined the impact of *Lactobacillus GG* on atopic disease was conducted by Kalliomäki et al. in 2001. Firstly, mothers with a family history of allergy were prenatally supplemented with *Lactobacillus GG* strains. Secondly, their infants received the same strain for the first six months of life. The research showed that the frequency of AD in the probiotic group was half that of the placebo group (23% vs. 46%), which took notice on the promising efficacy of probiotics in the prevention of allergic diseases [65]. Afterwards, Wang et al. investigated the effect of fermented milk containing *Lactobacillus paracasei-33* (LP-33) on the quality of life (QOL) of patients with AR. The results suggested that LP-33-fortified fermented milk intake for 30 days can effectively and safely improve AR patients’ QOL and may be used as an alternative treatment [66]. Researchers encouraged by the promising outcomes of mentioned studies decided to examine whether the bacteria strains’ efficacy depends on their activity. Peng et al. compared the impact of heat-killed LP-33 with its live strains in treating AR induced by house dust mite in human subjects. After the 30-day treatment, the outcomes were comparable. The heat-killed LP-33 was not inferior to the live variant, and both interventions improved the QOL of patients with AR compared with the placebo group [67]. Recently, Yan et al. collected 30 randomized controlled trials (RCTs) with probiotics as an intervention in AR and prepared their meta-analysis. It showed that compared with the placebo group, the Rhinitis Quality of Life (RQLQ) global score, RQLQ nasal score and Rhinitis Total Symptom Score (RTSS) for nasal symptoms were significantly improved after probiotic supplementation. However, there was no significant difference between the placebo and the probiotic group in the blood eosinophil count, RQLQ eye score, RTSS global score, RTSS eye score, and total and antigen-specific serum IgE levels. Noticeably, the vast majority of included studies used probiotics composed of *Lactobacillus* and *Bifidobacterium* strains. Most studies did not report any adverse effects. However, some studies lacked quantifiable data, and their outcomes were incomplete; thus, there is an urgent need to conduct more high-quality RCTs [68].

The mechanism of probiotics’ action in reducing the risk and improving the clinical course of AR is not fully understood. However, Kukkonen et al. showed that probiotic supplementation might be linked with a high secretion of mucosal IgA, which participates in antigen elimination [69]. Furthermore, the consumption of probiotics may reduce the secretion of antigen-specific IgE and Th2 cytokines (IL-4, IL-13) [70].

It is worth pointing out that the International Consensus Statement on Allergy and Rhinology: Allergic Rhinitis recommends considering probiotics as an adjuvant therapy for patients with AR due to their minimal harm and proven efficacy in improving symptoms [71]. 

### 5.2. Prebiotics

Prebiotics are specific dietary ingredients that affect the composition and activity of the gut microbiota. To become supplements, they must meet some inclusion criteria. Firstly, prebiotics must be resistant to gastric acidity, hydrolysis by enzymes and gastrointestinal absorption. Secondly, they should be fermented by intestinal microbiota and able to stimulate the growth of beneficial bacteria [72]. The most used prebiotics are lactitol, lactulose, inulin, lactosucrose, fructooligosaccharides, galactooligosaccharides and soy oligosaccharides [73]. They may be used as an alternative to probiotics or as a support for them. 

Only one study examined the prebiotics’ impact on AR adults with high IgE levels. For 52 weeks, patients received lactosucrose, and their serum IgE levels were measured. After a year of treatment, serum IgE levels (especially to pollen allergens) significantly decreased, accompanied by the relief of allergic symptoms [74]. Furthermore, Derakhshan et al. investigated the effect of dried Ma-al-Shaeer (a traditional, rich-in-fibre, Iranian medicine with a formulation based on barley) versus fexofenadine on adult AR patients. Enrolled participants received orally mentioned preparations twice a day for 14 consecutive days. The clinical course of AR was improved in both groups, whereas nasal congestion, post-nasal drip and headache scores were significantly decreased in the Ma-al-Shaeer group [75]. 

Prebiotics are widely used as supplementation to milk formula for infants. Arslanoglu et al. evaluated the protective effect of prebiotic oligosaccharides against allergy. In this RCT, healthy infants with a risk of atopy were fed prebiotic-supplemented or placebo-supplemented formula during the first six months of life. The follow-up period lasted for five years. The cumulative incidences of allergy manifestation were significantly lower in the prebiotic supplemented group. The intervention was particularly beneficial in allergic rhinoconjunctivitis and allergic urticaria prevention [76]. 

In a mouse model of allergy, 2’-fucosyllactose and 6’-sialyllactose stimulated IL-10 production and stabilized mast cells [77]. The study by Gourbeyre et al. showed that sensitized mice supplemented with prebiotics or not had similar levels of IgE, IgG1, IL-4, IL-17 and allergy symptoms. However, the levels of IgG2a, specific IgA, IL-10, TGF-β and IFN-γ were significantly higher in the prebiotic treated group. This suggests that, in the mouse model, the exposure to prebiotics during perinatal and postweaning periods induces the highest expression of biomarkers related to tolerance without affecting biomarkers related to allergy [78].

To conclude, there are still insufficient data regarding prebiotics use in the prevention and treatment of AR. Nevertheless, their ability to modulate cytokine release seems to be a new, promising approach to the treatment of allergic diseases.

### 5.3. Bacterial Lysates

BLs are immunomodulatory preparations consisting of antigens derived from respiratory tract pathogens. The most common are *Streptococcus pneumoniae, Haemophilus influenzae, Moraxella catarrhalis, Streptococcus pyogenes, Streptococcus viridans, Staphylococcus aureus, Klebsiella pneumoniae* and *Klebsiella ozaenae* [79]. The preparation can be obtained using chemical or mechanical lysis. Different production methods can result in different immune effects. BLs can be administered orally, intranasally and sublingually [80]. This review discusses oral administration due to its impact on the gut environment.

As mentioned before, BLs’ mechanism of action is based upon natural exposure to pathogen antigens and the following immune response. They activate DCs through Toll-like receptors, promoting antiviral cytokines release, NK cell activation, and the restoration of the Th1/Th2 balance. The in-depth mechanism of BLs’ action is described in our previous publication [81].

Only five studies investigated BLs’ impact on the AR course. Two of them used orally administered OM-85. Koatz et al. conducted an open-label, sequential study concerning the use of OM-85 on respiratory tract infection rates, primary disease exacerbation rates and symptom severity in patients with AR, asthma or chronic obstructive pulmonary disease. Patients received the preparation in three cycles consisting of 10 consecutive days of intake followed by a 20-day break. They showed that OM-85 therapy reduced the number of respiratory tract infections and AR exacerbations, and the severity of allergic symptoms in comparison to the previous year when patients received only standard optimized care. Moreover, an increase in serum and salivary IgA levels has been demonstrated [82]. To further expand the research, Meng et al. evaluated its clinical effects in 60 patients with perennial AR. Enrolled participants were administered with OM-85 following the same regimen as in the previously mentioned study. After the treatment, the OM-85 group presented a significant decrease in total nasal symptom score, itching score, nasal rhinorrhoea score, sneezing score and medication score. Furthermore, an increase in nasal IFN-γ and decreases in nasal IL-4 and IL-13 levels, and the number of eosinophils in nasal swabs were observed [83]. 

BLs do not affect the intestinal bacteria directly; however, the cytokines stimulated by them may affect the gut environment. Van Averbeke et al. suggest that host immunity influences the composition of gut microbiota. In a murine model, they characterized the humoral, cellular and cytokine immunity and associated alterations in the gut microbiota of naïve wild-type mice and mice with deficiencies in Th2 responses or both Th1 and Th2 responses. The research showed that wild-type mice were enriched in bacteria, which are able to stimulate beneficial SCFAs production. Furthermore, the Th1-leaning compared to the naïve wild-type mice presented less microbial diversity. These data suggest that alterations in the Th1/Th2 balance or complete ablation of Th1/Th2 responses can significantly alter gut microbiota composition and function [84].

### 5.4. Fecal Microbiota Transplantation

FMT is the procedure in which the healthy donor’s stool is transformed into faecal suspension and is administered to the patient’s intestine to re-establish the balance of the gut microbiota [85]. Currently, there are no studies concerning its use in AR treatment; however, it may be a promising way to restore the intestine bacteria composition. Potentially, it may be more effective than probiotics due to its significantly more abundant infused microorganisms and the ability to permanently colonize the gut [86].

This presumption is being upheld by the study by Mashiah et al., who investigated the effects of FMT in adult AD patients. It was shown that the Scoring Atopic Dermatitis Score (SCORAD) significantly decreased after FMT. Moreover, weekly topical usage of corticosteroids was diminished during the study and follow-up period. Metagenomic analysis of the gut microbiota showed a significant bacterial strain transmission from donors to patients. No adverse effects of the treatment were observed [87]. Nevertheless, there are still insufficient data concerning its use in allergic diseases; thus, more large sample studies are needed.

## 6. Conclusions

In summary, it seems highly probable that gut microbiota plays a role in the pathogenesis of AR. With ever-growing evidence published in that field, the hope for the new prevention and therapy of AR is growing in parallel. 

It is believed that immune system imbalance is linked to decreased gut microbiota composition. Prenatal, neonatal and early childhood bacteria colonization alters the gut environment and impacts the developing immune system. Thus, early-life disturbances in colonization may affect future host’s immunity and lead to allergy. However, probiotics, prebiotics and BLs seem promising therapeutic approaches to restore this imbalance.

In the future, it will probably be possible to identify patients with an exceptionally high allergy risk or even modulate intestinal microbiota effectively and permanently. Nevertheless, more rigorous and detailed trials are demanded to draw definitive conclusions.

## Data Availability

Not applicable.
